# Frontotemporal lobar degeneration complexity: atypical presentations and heterogeneous proteinopathies in five cases

**DOI:** 10.3389/fnins.2026.1774283

**Published:** 2026-02-26

**Authors:** Giulia Negro, Camilla Poloni, Valentina Medici, Chiara Calatozzolo, Alessandra Canazza, Riccardo Rocco Ferrari, Chiara Cutaia, Annalisa Davin, Tino Emanuele Poloni

**Affiliations:** 1 Ph.D. Program in Neuroscience, School of Medicine and Surgery, Università degli Studi di Milano-Bicocca, Milan, Italy; 2 Department of Neurology and Neuropathology, Fondazione Golgi Cenci, Milan, Italy; 3 Department of Neurology, ASST Ovest Milano Legnano Hospital, Milan, Italy; 4 Department of Translational Medicine, University of Eastern Piedmont, Novara, Italy; 5 Laboratory of Neurobiology and Neurogenetics, Fondazione Golgi Cenci, Milan, Italy; 6 Department of Dementia Care, ASP Golgi-Redaelli, Milan, Italy; 7 Department of Rehabilitation, ASP Golgi-Redaelli, Milan, Italy

**Keywords:** alpha-synuclein, Alzheimer’s disease, amyloid, dementia, FTLD, neuropathology, tauopathy, TDP-43

## Abstract

**Introduction:**

Frontotemporal lobar degeneration (FTLD) encompasses heterogeneous clinical syndrome within the frontotemporal spectrum, where clinicopathological associations may be misleading. This case series illustrates clinicopathological variability and mismatches.

**Methods:**

A retrospective case series was conducted within the brain donation program at the Golgi Cenci Foundation. Cases presenting at onset with a frontotemporal-spectrum phenotype, longitudinal clinical data, and post-mortem neuropathological characterization were included.

**Results:**

Five cases (mean age at onset 65.4 years) were clinically diagnosed with major neurocognitive disorder due to frontotemporal dementia (FTD). Neuropathological examination revealed clinicopathological heterogeneity: two cases showed FTLD-TDP-A associated with GRN mutations, including a classic case and one with posterior (parieto-occipital) involvement; one non-fluent variant primary progressive aphasia (nfvPPA) case demonstrated FTLD-TDP-A with multiple co-pathologies; one semantic-variant-like case was driven by high Alzheimer’s disease neuropathological changes; and one behavioral variant FTD-like case corresponded to frontal-variant Alzheimer’s disease (fvAD) with extensive mixed pathology, including Lewy body disease, LATE-NC, and vascular pathology.

**Discussion:**

Findings indicate that clinical phenotypes are more influenced by the anatomical distribution of pathology than by the specific molecular substrate. Frequent coexisting proteinopathies and asymmetric involvement contribute to phenotypic variability, reinforcing the role of neuropathological examination of both hemispheres for accurate clinicopathological correlations and definitive etiological diagnosis.

## 1 Introduction

The term frontotemporal lobar degeneration (FTLD) refers to a group of progressive neurodegenerative disorders that are clinically, pathologically and genetically heterogeneous, but share selective frontal and/or temporal cortical atrophy. FTLD is a common cause of early-onset dementia, with an average age of onset between 45 and 65 years. Among its clinical subtypes, frontotemporal dementia (FTD) is mainly characterized by behavioral symptoms, while aphasic variants comprehend the non-fluent/agrammatic variant primary progressive aphasia (nfvPPA) and the semantic variant (svPPA). Besides behavioral and language dysfunctions, FTLD frequently comprises motor manifestations, including atypical parkinsonian syndromes and motor neuron disorders (Bang et al., 2015; Rabinovici and Miller, 2010).

Although many FTLD cases are sporadic, up to 50% show a positive family history. The most frequent genetic causes of familial FTD are mutations in chromosome 9 open reading frame (C9orf72), microtubule associated protein tau (MAPT) and progranulin gene (GRN), which together account for approximately 60% of inherited cases (Olszewska et al., 2016).

The most prevalent clinical presentation is the behavioral variant of FTD (bvFTD), accounting for approximately half of FTLD cases (Johnson et al., 2005). Typical symptoms include disinhibition, loss of empathy, apathy, changes in eating habits, stereotyped behaviors, and executive dysfunctions affecting planning, cognitive flexibility and judgment (Boeve et al., 2022; Rascovsky et al., 2011). Neuroimaging studies show involvement of orbitofrontal, fronto-insular, dorsolateral prefrontal, anterior cingulate and anterior temporal cortices with regional patterns of atrophy correlating with specific behavioral symptoms (Rabinovici and Miller, 2010).

nfvPPA is characterized by apraxia of speech, agrammatism, progressively reduced verbal output, and impaired grammatical comprehension with relative preservation of single word meaning (Boeve et al., 2022; Gorno-Tempini et al., 2011; Thompson and Mack, 2014). Neuroimaging studies typically show left perisylvian atrophy, involving the frontal operculum, premotor and supplementary motor areas and anterior insula (Rabinovici and Miller, 2010).

Instead, svPPA is characterized by impaired single-word comprehension and naming deficits, reflecting a loss of semantic knowledge, while sentence-level comprehension is relatively preserved in early stages. Speech remains fluent but semantically empty, often appearing structurally intact despite reduced informational content (Gorno-Tempini et al., 2011). As the disease progresses, subjects develop severe anomia and comprehension deficit even for highly familiar concepts (Meteyard and Patterson, 2009). Neuroimaging reveals predominant atrophy of the anterior temporal lobe (Rabinovici and Miller, 2010).

FTLD is neuropathologically heterogeneous, including tauopathies (FTLD-TAU), TAR DNA-binding protein 43 (TDP-43) pathologies (FTLD-TDP), and, less commonly, Alzheimer’s disease (AD)-related pathology (Bang et al., 2015; Gorno-Tempini et al., 2011; Rabinovici and Miller, 2010; Rascovsky et al., 2011). In this complex clinical and anatomical setting, neuropathological characterization is required to define the nature and distribution of pathological protein aggregates and to support clinicopathological correlations.

This case series presents a selection of neuropathologically examined cases from our cohort, showing clinical phenotypes within the frontotemporal spectrum at disease onset. This series aims to illustrate the heterogeneity within the FTLD spectrum and the importance of neuropathological characterization to clarify the underlying pathophysiology of these disorders.

## 2 Materials and methods

The results of this study are reported according to the Case Report Checklist CARE guidelines for case series (Gagnier et al., 2013). This retrospective case-series was compiled using longitudinal clinical and neuropathological data collected at the Golgi Cenci Foundation (Abbiategrasso, Italy) within the brain donation program in the frame of the InveCe.Ab cohort (Invecchiamento Cerebrale Abbiategrasso study), which currently comprises 60 cases (Guaita et al., 2013). The clinical neurocognitive diagnosis was formulated based on the Diagnostic and Statistical Manual of Mental Disorders, Fifth Edition (American Psychiatric Association [APA], 2013).

Eligible cases met the following criteria: (I) a clinical phenotype within the frontotemporal spectrum disorder, including behavioral or language presentations (bvFTD, svFTD, nfvPPA), diagnosed according to established clinical criteria (Gorno-Tempini et al., 2011; Rascovsky et al., 2011); (II) availability of a complete neuropathological characterization; (III) provision of written informed consent for brain donation. Cases were included without additional selection beyond eligibility criteria.

During life, brain donors underwent standardized neurological and neuropsychological assessments as established in the Abbiategrasso Brain Bank protocol (Poloni et al., 2020). After death, brain tissue was processed following the same protocol: alternate coronal sections of the cerebral hemisphere, brainstem, and cerebellum were either snap-frozen at −80 °C or fixed in phosphate-buffered formalin and subsequently paraffin-embedded. Paraffin sections then underwent the histological and immunohistochemical (IHC) staining procedures required for diagnostic assessment. Neuropathological assessment was performed following international consensus criteria, including Alzheimer-type pathology (Braak et al., 2006; Mirra et al., 1991; Montine et al., 2013; Thal et al., 2002), LTS (Beach et al., 2009), TDP-43 proteinopathies (Josephs et al., 2014; Mackenzie et al., 2011; Nelson et al., 2019), hippocampal sclerosis (HS) (Rauramaa et al., 2013) and cerebrovascular pathology (Deramecourt et al., 2012; Skrobot et al., 2016). A detailed description of the tissue processing steps, histological and IHC staining, and diagnostic consensus criteria is reported in the Abbiategrasso Brain Bank protocol (Poloni et al., 2020). Neuropathological diagnoses were formulated based on the type and anatomical distribution of lesions and assigned in accordance with validated diagnostic criteria.

Ethical approval for all study procedures was obtained from the Ethics Committee of the University of Pavia (Committee report 3/2009), within the framework of the InveCe.Ab project (Guaita et al., 2013). Written informed consent for clinical evaluation, brain donation, neuropathological examination, and research use of data was obtained from all donors in accordance with the ethical principles governing the brain donation program of the Fondazione Golgi Cenci (Klioueva et al., 2015). All procedures were conducted in full compliance with the Declaration of Helsinki and subsequent amendments, and oversight by “Federazione Alzheimer Italia” ensured adherence to relevant ethical and professional standards.

## 3 Results

Of our cohort of 60 cases, five cases met the eligibility criteria for inclusion in the case series. Of these, two were women and three were men. The mean age at onset was 65.4 years (SD ± 8.1), and the mean age at death was 71.8 years (SD ± 7.4). At the first clinical evaluation, all five cases had a clinical diagnosis of major-NCD due to FTD; the diagnosis of one case was later revised to a possible frontal variant Alzheimer’s disease (fvAD), according to a subsequent biomarkers assessment.

A summary of clinical and neuropathological profiles of the five cases is reported in Tables 1, 2.

**TABLE 1:** Summary of the clinical profiles of the five cases.

Case	Sex	Age at onset	Age at death	Symptoms at onset	First clinical diagnosis	Clinical course	Last clinical diagnosis	Family history	Genetic mutation
Case 1	M	55	63	Apathy; social withdrawal; disinhibition; hyperorality	bvFTD	Worsening behavioral disturbances; progressive non-fluent aphasia to global aphasia; progressive parkinsonism; final stage with spastic tetraparesis	bvFTD	+	GRN c.813_816delCAT Not performed
Case 2	F	59	65	Disorientation in familiar places; visuospatial deficits (left-sided); alexia; mild behavioral symptoms	PCA/bvFTD	Behavioral disturbances (impulsivity, delusions, hyperorality, sweet cravings, mood deflection, verbal stereotypies); reduced, logopenic speech; progressive parkinsonism; late-stage catatonia	bvFTD	+	GRN Thr272fs APOE ε3/ε3
Case 3	F	71	76	Non-fluent speech; preserved simple comprehension	nfvPPA/CBS	Progressive language deterioration to mutism; bucco-facial apraxia; worsening memory; behavioral disturbances; late-stage right-sided parkinsonism and dystonia	nfvPPA	+	Not performed APOE ε3/ε3
Case 4	M	74	80	Fluent aphasia with comprehension and naming deficits; spatial disorientation; behavioral disturbances	svFTD	Worsening language with severe comprehension loss; behavioral disturbances; late-stage rigid-hypokinetic parkinsonism	svFTD	−	Not performed APOE ε3/ε3
Case 5	M	68	75	Apathy, abulia, loss of work interest; logopenic language impairment	bvFTD	Worsening memory, language and behavioral disturbances; spatial disorientation; late-stage parkinsonism	fvAD	−	Not performed APOE ε3/ε4

Overview of the demographic variables, initial presenting symptoms, key clinical progression features, final clinical diagnosis, family history suggestive of neurodegenerative disease, and results of genetic testing. bvFTD, behavioral variant frontotemporal dementia; PCA, posterior cortical atrophy; nfvPPA, non-fluent variant primary progressive aphasia; CBS, corticobasal syndrome; svFTD, semantic variant frontotemporal dementia; fvAD, frontal variant Alzheimer’s disease.

**TABLE 2:** Neuropathological profile of the five cases.

Case	Primary neuropathological diagnosis	Asymmetry	Additional proteinopathies	SVD	CAA	HS
Case 1	FTLD-TDP type A	Present	Absent	+	Absent	Present
Case 2	FTLD-TDP type A	Present	Low ADNC (A1B0C0)	+	+	Present
Case 3	FTLD-TDP type A	Present	Low ADNC (A1B1C0) Incidental LBD	++	+	Present
Case 4	Alzheimer’s disease (high ADNC: A3B3C3)	Present	Absent	+	+++	Present
Case 5	Alzheimer’s disease (high ADNC: A3B3C3)	Absent	Limbic LBD (Beach IIb) LATE-NC stage 1	+	++	Present

Overview of the main neuropathological findings for each case, including the primary neuropathological diagnosis, the asymmetrical distribution of proteinopathies, and the presence or absence of Alzheimer-type changes, Lewy-type α-synuclein pathology, pTDP-43 proteinopathy, cerebrovascular pathology, and hippocampal sclerosis. Alzheimer-type pathology is reported using the NIA-AA “ABC” score—A: Amyloid; B: Braak; C: CERAD for neuritic plaques (Montine et al., 2013). The intensity of each pathological change is indicated using the following symbols: + mild, ++ moderate, +++ severe. ADNC, Alzheimer’s disease neuropathologic change; CAA, cerebral amyloid angiopathy; SVD, small-vessel disease; HS, hippocampal sclerosis; LBD, Lewy body disease; LATE, limbic-predominant age-related TDP-43 encephalopathy; FTLD, frontotemporal lobar degeneration.

### 3.1 Case presentation

A case-by-case description of patients included in the study is presented below.

### 3.2 Case 1

The first patient was a man who first presented at age 55 with prominent behavioral disturbances, including apathy, social withdrawal, aggressivity, disinhibition and hyperorality. One year later, he developed progressive non-fluent aphasia, along with executive dysfunction and apraxia.

MRI and 18F-fluorodeoxyglucose PET (18F-FDG-PET) scans performed the same year showed fronto-temporal atrophy, predominantly left side, with corresponding left fronto-temporal hypometabolism. Targeted genetic testing revealed a pathogenic GRN mutation (c.813_816delCAT). A diagnosis of bvFTD was established according to current diagnostic criteria.

Over the following three years from symptom onset, his behavioral disturbances worsened, with the emergence of obsessive behaviors, psychomotor agitation, wandering, insomnia and hyperphagia. On neuropsychological evaluation, repeated Mini-Mental State Examination (MMSE) assessments remained within the normal rages (education level: 18 years). His medical history included arterial hypertension and a major depressive episode at age 22.

At age 60, he experienced further neurological deterioration, with the development of severe right-sided hemiparkinsonism and pseudobulbar syndrome. The last neurological examination, performed at age 63, revealed complete functional dependence, global aphasia, severe dysphagia, double incontinence and prominent frontal release signs. Motor examination showed severe spastic tetraparesis with mixed hypertonia, more pronounced in the upper limbs, and advanced sarcopenia. The patient died at age 63 years due to complications of end-stage dementia.

Family history was notable for FTD-related disorders, including a cousin with nfPPA due to a GRN mutation, and a father and maternal aunt with unspecified dementia without further diagnostic evaluation. Apolipoprotein E (APOE) genotyping was not performed.

**FIGURE 1:**
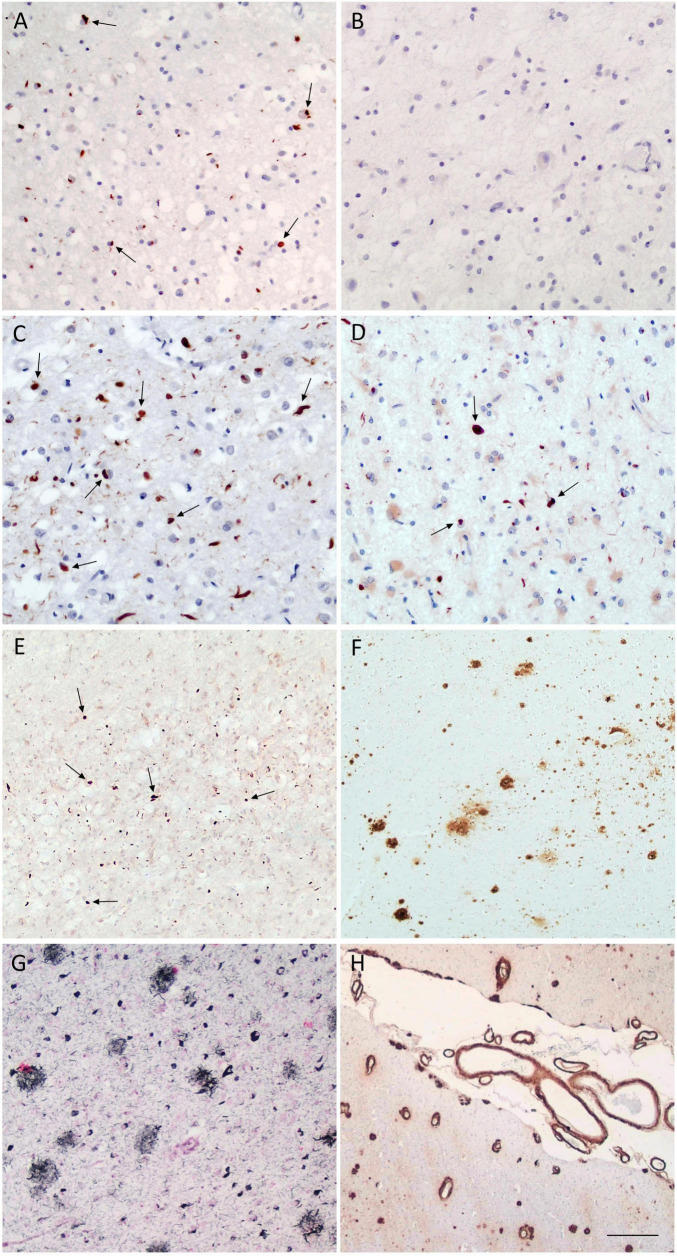
FTLD-TDP pathology and associated co-pathologies in Cases 1–4. Representative immunohistochemical findings from cortical regions sampled Cases 1–4. Case 1 (FTLD-TDP-A): pTDP-43-positive NCIs (arrows) and DNs in the frontal cortex **(A)**, with absence of pTDP-43 pathology in the parietal cortex **(B)**. Case 2 (FTLD-TDP-A): widespread pTDP-43-positive NCIs (arrows) and DNs involving both frontal **(C)** and parietal cortices **(D)**. Case 3 (FTLD-TDP-A with low ADNC): pTDP-43-positive NCIs (arrows) in the frontal cortex **(E)**, together with amyloid-β deposits in the same region (Thal phase 1) consistent with low ADNC in the same region **(F)**. Case 4 (high ADNC): neuritic plaques in neocortex (CERAD 3) **(G)** and severe leptomeningeal and parenchymal cerebral amyloid angiopathy **(H)**. Scale bars: **(A,C,D)** = 45 μm; **(B)** = 77 μm; **(E,G)** = 90 μm; **(F)** = 224 μm; **(H)** = 325 μm. FTLD, frontotemporal lobar degeneration; pTDP-43, phosphorylated TAR DNA-binding protein 43; NCI, neuronal cytoplasmic inclusion; DN, dystrophic neurite.

Post-mortem neuropathological examination revealed severe bilateral atrophy of the fronto-temporal poles, along with severe hippocampal atrophy. Microscopic analysis showed severe neuronal loss with spongiosis in cortical layer II, and gliosis in the fronto-temporal cortex, hippocampus and basal ganglia, with milder parietal involvement. IHC studies demonstrated predominant FTLD-TDP type A pathology, characterized by many neuronal pTDP-43 cytoplasmic inclusions (NCIs), neuronal intranuclear inclusions (NIIs), and dystrophic neurites (DNs) in layer II of the fronto-temporal cortex (Figure 1A), and in the hippocampal dentate gyrus; parietal cortex was spared (Figure 1B). No amyloid-β, tau or α-synuclein pathology was identified. Cerebrovascular pathology was mild (VCING score 1; Deramecourt vascular score 3). HS was also present (Rauramaa stage 4). Overall, the primary neuropathological diagnosis was FTLD-TDP-A with associated with HS.

### 3.3 Case 2

The second patient was a woman whose symptoms began at age 59 with disorientation in familiar environments and difficulty navigating space due to space perception deficit. She had left-sided visual field deficit and reading difficulties (alexia). Several months later, behavioral symptoms emerged including mood deflection, delusions, wandering, hyperorality, sweet cravings, loss of impulse control, and verbal stereotypies. She subsequently developed reduced speech output with logopenic features and echolalia, while memory impairment remained relatively mild throughout the disease course. Her medical history was unremarkable, and her level of education was 13 years.

At age 61, brain MRI showed diffuse cortical and subcortical atrophy, predominantly affecting the right temporo-parieto-occipital regions, with enlargement of the right lateral ventricle, trigone, and occipital horn, and thinning of the right optic radiation, consistent with the contralateral visuospatial deficit. A small right parietal meningioma was also identified but deemed incidental.

Despite a picture of posterior cortical atrophy (PCA), the following clinical course oriented toward a diagnosis of bvFTD, confirmed by genetic testing that revealed a heterozygous pathogenic GRN Thr272fs variant.

At age 62, she developed motor symptoms, initially characterized by a bilateral rigid-hypokinetic parkinsonian syndrome with postural instability and recurrent falls, later evolving into catatonic features. In the later stages, behavioral abnormalities progressed to severe apathy. She died at age 65 due to complications related to end-stage dementia.

Family history was notable for multiple affected relatives. Her mother developed behavioral symptoms at age 69 and died at age 89. A maternal uncle had Parkinson’s disease and died at age 55. A maternal aunt developed dementia in her nineties but did not undergo diagnostic assessment. Two brothers were reportedly affected by memory impairment, behavioral disturbances, and motor symptoms; one of them manifested dementia and parkinsonism but tested negative for the GRN mutation. APOE genotyping revealed an ε3/ε3 genotype.

Post-mortem neuropathological examination revealed severe generalized cortical atrophy with right hemispheric predominance. Microscopically, marked neuronal loss with spongiosis in cortical layer II, and gliosis involved the frontal cortex, hippocampal cornus ammonis 1 (CA) and subiculum, substantia nigra (SN), locus coeruleus, and cranial nerve nuclei X and XII. Cerebrovascular pathology was mild (VICING score 1; Deramecourt vascular score 7/20). IHC analysis demonstrated widespread pTDP-43 pathology, with numerous NCIs and DNs predominantly affecting the frontal, temporal, insular, and parietal cortices (Figures 1C, D), as well as the amygdala, thalamus, hippocampal CA1 and subiculum of the right hemisphere. Additional mild pTDP-43 pathology was found in the basal ganglia and in the occipital cortex. The morphology and distribution were consistent with FTLD-TDP type A. HS was present (Rauramaa stage 3). Tau pathology was minimal (Braak stage I), and no amyloid-β deposits were identified. Overall, the neuropathological diagnosis was FTLD-TDP-A associated with HS.

### 3.4 Case 3

The third patient was a woman who first presented at age 71 with progressive language impairment characterized by non-fluent speech with anomia, agrammatism, and preserved comprehension of simple tasks. She subsequently developed progressive verbal memory deficits with bucco-facial apraxia and behavioral disturbances, including hyperorality, perseverations, sweet cravings, disinhibition, and apathy. A brain MRI performed at age 73 revealed asymmetrical left-sided atrophy, predominantly involving the frontal lobe. A clinical diagnosis of nfvPPA was made according to established criteria. Medical history was otherwise insignificant, apart from arterial hypertension and previous hip prosthesis surgery.

At age 74, her clinical condition further deteriorated, with partial functional dependence. Language impairment worsened until mutacism, with partial preservation of comprehension. At that time, the MMSE score was 21 (education level: 8 years).

The last neurological assessment, performed at age 75, showed complete functional dependence, global aphasia, dysphagia, double incontinence, severe apathy, inertia and frontal release signs. Motor examination showed limitation of vertical gaze and severe right-sided hemiparkinsonism with dystonia. EEG demonstrated diffuse centro-anterior slowing with preserved posterior dominant rhythm (9 Hz). The patient died at age 76 due to complications related to end-stage dementia.

Family history was positive for dementia: her father developed cognitive impairment at age 74, followed by a pseudobulbar syndrome with progressive motor deficits leading to bed confinement. Her paternal sister died at age 80 from an undocumented neurological disease. Genetic testing was declined by relatives. APOE genotyping showed an ε3/ε3 genotype.

Post-mortem neuropathological examination showed asymmetrical atrophy predominantly involving the left fronto-temporal lobe, mammillary body, and caudate nucleus, together with pallor of the SN. Microscopic evaluation revealed severe neuronal loss and gliosis in the SN, hippocampal formation, left caudate nucleus, and fronto-temporal cortex. Cerebrovascular pathology demonstrated moderate small-vessel disease with leptomeningeal cerebral amyloid angiopathy (CAA; VCING 1; Deramecourt vascular score 7/20). IHC revealed a pTDP-43 proteinopathy, with asymmetric NCIs and short DNs predominantly affecting the left insular and frontal cortices, with spongiosis in cortical layer II (Figure 1E), as well as the basal ganglia. Additional pTDP-43 inclusions were observed in the left temporo-parietal cortex, amygdala, hippocampal formations, albeit with milder severity. In the frontal white matter, frequent oligodendroglial cytoplasmic inclusions (CGIs) and pTDP-43 threads were observed. The overall distribution was consistent with FTLD-TDP type A. Alzheimer’s disease neuropathological changes (ADNC) were also identified, characterized by Aβ deposition consistent with Thal phase 1 (Figure 1F), mild neurofibrillary tangles (NFTs) and neuropil threads in the amygdala, entorhinal and temporo-occipital cortices (Braak stage I). Focal subpial aging-related tau astrogliopathy (ARTAG) was present in the mesial temporal region. No neuritic plaques were detected (CERAD 0). According to NIA-AA criteria, the overall ADNC were classified as low ADNC (A1B1C0). Lewy-type α-synucleinopathy (LTS) was identified, mainly involving the nucleus basalis of Meynert, with rare incidental brainstem inclusions. HS was noted (Rauramaa stage 4). Overall, the primary neuropathological diagnosis was FTLD-TDP-A with multiple co-pathologies, including incidental Lewy body pathology, low ADNC, and mild-to-moderate small-vessel disease.

### 3.5 Case 4

The fourth patient was a man who first presented at age 74 with progressive language impairment characterized by fluent aphasia with comprehension and naming deficits. Episodes of spatial disorientation and apraxia were also reported. Concomitantly, he developed behavioral disturbances, including wandering, verbal and physical aggressivity, irritability, delusions, stereotyped behaviors, and elopement attempts. The assessment of other cognitive domains was hindered by the severity of the language disorder. No family history of neurodegenerative disease was reported; APOE genotyping revealed an ε3/ε3 genotype. His medical history included pulmonary tuberculosis and arterial hypertension.

Repeated MMSE evaluations showed progressive cognitive decline, from a score of 19 at age 74 to 4 at age 76 (education level: was 8 years). A brain CT performed at age 76 revealed predominant asymmetric temporal lobe atrophy. Based on the clinical presentation and neuroimaging, a diagnosis of svFTD was established.

The last neurological assessment, performed at age 78, showed complete functional dependence, severe fluent aphasia with marked comprehension deficits, reduced spontaneous speech with verbal stereotypies, and persistent behavioral disturbances. He was no longer testable with the MMSE. Motor examination revealed rigid-hypokinetic parkinsonism with postural instability and frontal release signs. He died at age 80 due to complications of end-stage dementia.

At post-mortem neuropathological examination, diffuse neocortical and allocortical atrophy was observed, together with pallor of the SN. Microscopic evaluation revealed severe neuronal loss in the hippocampal formation (CA1, subiculum) and in the locus coeruleus, with moderate temporo-parietal cortical involvement and gliosis. IHC demonstrated predominant Alzheimer-type pathology, with widespread amyloid-β plaques in the allocortex and neocortex (Thal phase 4). Tau pathology consisted of NFTs and neuropil threads in the hippocampi, temporal, insular, parietal, and focally occipital cortices (Braak stage V); the CERAD neuritic plaque score was 3 (Figure 1G). According to NIA-AA criteria, the overall ADNC were classified as high (A3B3C3). Pathological changes were more severe in the left temporo-parietal cortex and temporal pole. No pTDP-43 or α-synuclein pathology was detected. Mild small-vessel disease was present (VCING score 1; Deramecourt vascular score 7), together with severe leptomeningeal and parenchymal CAA and moderate capillary CAA (Figure 1H). HS was present (Rauramaa stage 4). The neuropathological primary diagnosis was consistent with high ADNC with HS, accompanied by mild small-vessel disease and widespread CAA.

### 3.6 Case 5

The fifth patient was a man who first presented at age 68 with behavioral disturbances, including apathy alternating with agitation due to delusions. Progressively, he developed loss of occupational interest and abulia. One year later, he developed logopenic language impairment and memory deficits. An initial diagnosis of bvFTD was suggested according to established criteria. He had a high cognitive reserve (education level: 17 years), and the MMSE score was 28/30. His medical history included arterial hypertension, dyslipidemia, and hyperhomocysteinemia with MTHFR mutations (C677T and A1298C). No family history of neurodegenerative disease was reported. APOE genotyping revealed an ε3/ε4 genotype.

A brain MRI performed at age 69 revealed symmetrical temporal lobe atrophy and lacunar infarcts in the external capsule and basal ganglia, while a 18F-FDG-PET showed fronto-temporal and anterior cingulate hypometabolism, with additional involvement of bilateral inferior parietal lobules. Cerebrospinal fluid (CSF) biomarkers of neurodegeneration showed: total tau 427 pg/mL (normal < 500), phosphorylated tau 61 pg/mL (normal < 61), and Aβ42 373 pg/mL (normal > 500). EEG showed a posterior dominant rhythm of 9–10 Hz, with intermittent bifrontal slowing (4–6 Hz). Following the diagnostic investigations a clinical diagnosis of possible fvAD was made.

From age 71 onward, he experienced progressive cognitive deterioration with spatial disorientation, worsening memory deficits, and further language impairment. Serial MMSE assessments demonstrated gradual decline until age 73, when testing was no longer feasible due to severe aphasia. Behavioral symptoms also worsened, including hyperactivity, irritability, aggressivity, sweet cravings, wandering, sundowning agitation, delusions, and sleep-wake cycle disruption. At age 72, he developed extrapyramidal signs, including hypomimia, hypokinesia, and tremor, which progressed to a severe generalized rigid-hypokinetic parkinsonian syndrome. An EEG performed at age 74 showed marked deterioration, with background slowing (5 Hz) and bilateral delta activity in the fronto-temporal regions. The patient died at age 75 due to end-stage dementia.

**FIGURE 2:**
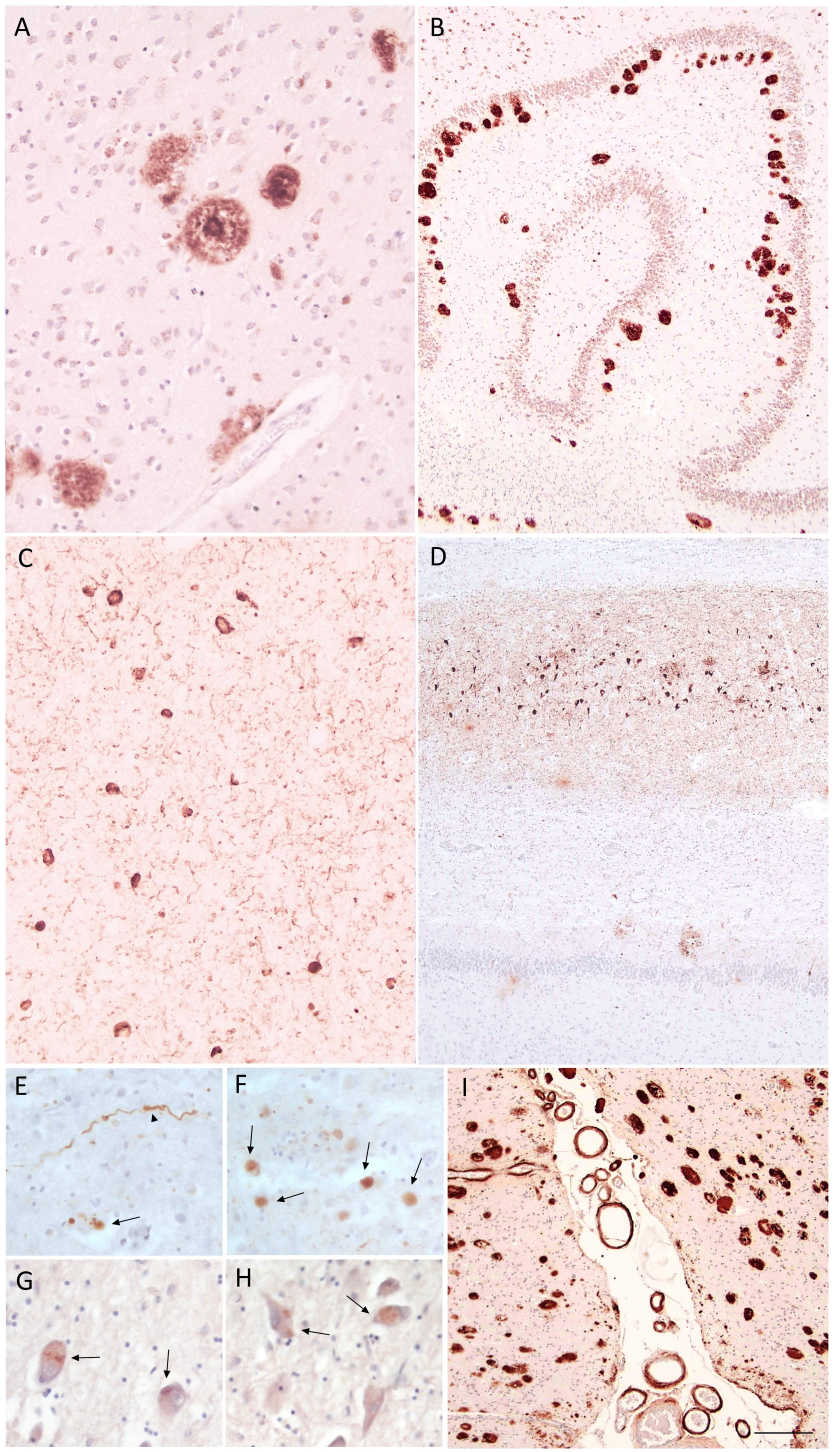
Alzheimer neuropathological changes and co-pathologies in Case 5. Neuropathological findings illustrating mixed dementia in Case 5, clinically diagnosed with fvAD. Amyloid-β immunostaining shows dense-core plaques in the frontal cortex **(A)** and widespread deposition in the hippocampal formation **(B)** (Thal phase 5); pTau immunostaining reveals abundant NFTs and neuropil threads in the frontal cortex **(C)** and hippocampal regions **(D)**, consistent with high ADNC; α-Synuclein immunostaining demonstrates Lewy-type pathology in the amygdala with dystrophic neurites (arrowhead) and Lewy bodies (arrows) **(E,F)**, consistent with limbic-predominant Lewy body disease (Beach IIb); pTDP-43 immunoreactivity shows NCIs (arrows) in the amygdala **(G,H)**, consistent with LATE-NC stage 1; moderate parenchymal and leptomeningeal cerebral amyloid angiopathy is shown in **(I)**. Scale bars: **(A,C)** = 80 μm; **(B,D,I)** = 220 μm; **(E–H)** = 43 μm. NCI, neuronal cytoplasmic inclusion; neurofibrillary tangles, NFT; pTDP-43, phosphorylated TAR DNA-binding protein 43; LATE-NC, limbic-predominant age-related TDP-43 encephalopathy neuropathological change.

At post-mortem neuropathological examination, bilateral moderate frontal, temporal, and right cingulate atrophy was observed, together with pallor of the SN. Microscopic assessment revealed neuronal loss and gliosis in the frontal cortex, hippocampal CA1 and subiculum, SN, locus coeruleus, and cranial nerve nuclei X and XII. IHC analysis demonstrated Alzheimer-type pathology with extensive amyloid plaques in neocortex, allocortex (Figures 2A, B) and basal ganglia, and mild-to-moderate involvement of the brainstem (Thal stage 5). Widespread NFTs, neuropil threads and neuritic plaques were detected in both neocortical and allocortical regions (Figure 2C, D; Braak stage V-VI; CERAD 3). According to NIA-AA criteria, the overall ADNC were classified as high (A3B3C3). Severe limbic-predominant LTS was identified (Beach score IIb), particularly in amygdala (Figures 2E, F), along with pTDP-43 pathology in amygdala (Figures 2G, H), which is consistent with limbic-predominant age-related TDP-43 encephalopathy-neuropathological changes (LATE-NC stage 1). HS was present (Rauramaa stage 4). Mild small-vessel disease was present (VCING score 1; Deramecourt vascular score 1/20), together with moderate parenchymal and leptomeningeal CAA (Figure 2I). Overall, the neuropathological diagnosis was mixed dementia, characterized by high ADNC, limbic-predominant Lewy body disease, and LATE-NC stage 1, HS and moderate CAA.

## 4 Discussion

This case series includes emblematic examples of the heterogeneity of FTLD, highlighting the variability of clinical presentations and the range of underlying proteinopathies encountered in the frontotemporal dementia spectrum.

The first case represents the most “classical” presentation within the series, with an early behavioral syndrome consistent with bvFTD associated with a pathogenic GRN mutation, and a neuropathological diagnosis of FTLD-TDP-A. Neuroimaging revealed asymmetric atrophy and hypometabolism, and the patient developed aphasia and hemiparkinsonism, features classically associated with tauopathies. However, pTDP-43 pathology may also be associated with aphasia and parkinsonism (Mackenzie et al., 2011; Spinelli et al., 2017). Moreover, hemispheric asymmetry is a well-recognized feature of GRN-associated FTLD, described as a characteristic rather than an exception (Borrego-Ecija et al., 2024). Our findings corroborate these observations. This was the only case in the series with a relatively typical bvFTD presentation, whereas the remaining cases showed atypical phenotypes, reflecting that non-canonical presentations are common within the FTLD spectrum.

In the second case, the clinical presentation was characterized by predominant posterior and right-sided brain involvement, with early visuospatial disorientation and left-sided visuo-perceptual deficits, followed by behavioral symptoms and logopenic language impairment, while memory remained preserved. Although a PCA phenotype could initially be considered, current consensus classification requires the absence of early behavioral and language disturbances (Crutch et al., 2017), making this diagnosis unlikely. The appearance of behavioral symptom, and the subsequent identification of a pathogenic GRN Thr272fs mutation allowed a diagnosis of probable bvFTD with atypical onset. Rare bvFTD cases with posterior-predominant involvement have been reported, particularly in genetically determined forms associated with C9orf72 or GRN mutations (Caroppo et al., 2015; Floris et al., 2012), often accompanied by hemispheric asymmetry (Borrego-Ecija et al., 2024; Rohrer, 2012). Neuropathology confirmed asymmetric FTLD-TDP-A with an posterior distribution extending to parieto-occipital regions. This case highlights the phenotypic and anatomical heterogeneity of GRN-associated FTLD, and illustrates that posterior cortical involvement, although uncommon, can occur within the FTLD spectrum.

The third case presented with nfvPPA, associated with bucco-facial apraxia and behavioral disturbances, followed by rapidly progressive right-sided hemiparkinsonism with dystonia. Neuroimaging showed predominant left frontal involvement. While this presentation would classically suggest a nfvPPA with cortico-basal syndrome associated with an underlying tauopathy, neuropathology demonstrated predominant FTLD-TDP-A with extensive fronto-temporal and limbic involvement. Although less frequent, FTLD-TDP-A has been reported in nfvPPA (Mackenzie et al., 2011; Spinelli et al., 2017). Additional ADNC was detected, a finding previously described in nfvPPA (Spinelli et al., 2017). Another notable feature was the relatively late age at onset with a rapid disease progression. Older age increases the likelihood of amyloid co-pathology, and FTLD cases with mixed neuropathological changes tend to present later than “pure” forms (Bergeron et al., 2018; Seo et al., 2018). Unlike previous reports of longer disease duration in FTLD-TDP-A with co-pathology (Gefen et al., 2018), this case showed rapid evolution, underscoring the potential detrimental impact of co-pathologies and the variability of individual disease trajectories.

The fourth patient resembled svPPA, a syndrome most commonly associated with FTLD-TDP-C (Mackenzie et al., 2011). However, neuropathological examination revealed high ADNC, with predominant left parietal, anterior and mesial temporal involvement, without pTDP-43 pathology. Similar clinicopathological mismatches have been reported, as AD can rarely present with a semantic dementia-like phenotype and asymmetric involvement (Chow et al., 2010; Davies et al., 2005; Whitwell et al., 2019). This case supports prior reports, confirming that AD may rarely present with a svPPA clinical syndrome when neurodegeneration preferentially targets semantic networks. Thus, the clinical phenotype appears to depend more on anatomical distribution than on protein type, which cannot be reliably inferred from phenotype alone. Alongside neurodegenerative causes, an FTD-like phenotype may also reflect non-degenerative and sometimes treatable conditions, which should be considered in the differential diagnosis (Desmarais et al., 2021; Mez et al., 2013; Sugiyama et al., 2022). Finally, behavioral disturbances observed in this case may also be consistent with AD, where agitation, aggressivity, wandering, and delusions are frequently reported as a consequence of limbic involvement (Cerejeira et al., 2012; Negro et al., 2025).

The fifth patient initially presented with behavioral symptoms mimicking bvFTD, supported by 18-FDG-PET evidence of fronto-temporal and anterior cingulate hypometabolism. The subsequent development of logopenic aphasia and memory deficits suggested a more diffuse process, prompting further investigations, including CSF biomarkers consistent with AD. Neuropathology confirmed high ADNC with extensive frontal involvement which, along with severe limbic-predominant LTS, provided a plausible substrate for behavioral and parkinsonian features observed (Beach et al., 2009). Additional LATE-NC stage 1 and vascular pathology further highlighted the frequent coexistence of multiple proteinopathies in AD, particularly in cases with severe behavioral symptoms (Negro et al., 2025; Nelson et al., 2023). Although fvAD is typically characterized mainly by executive dysfunction, severe behavioral symptoms may occur in the presence of extensive frontal involvement and coexisting proteinopathies (Ossenkoppele et al., 2015). This case highlights the importance of longitudinal clinical follow-up and biomarker integration to improve diagnostic accuracy. Behavioral assessment in suspected FTD is often non-standardized, with potential delayed diagnoses; structured instruments such as the Mild Behavioral Impairment Checklist, the Frontal Systems Behavior Scale or the Frontal Behavioral Inventory can help mitigate this gap (Ismail et al., 2017; Kertesz et al., 2000; Malloy et al., 2007; Zapata-Restrepo et al., 2021). Further, functional imaging, such as diffusion MRI/DTI or FDG-PET, holds promise for improving syndromic and etiologic discrimination (Chandrasekar et al., 2025; Hoffmann, 2013; Santillo et al., 2013). However, definitive diagnosis ultimately relies on neuropathological examination, which often reveals clinically relevant pathologies.

Across the series, the clinicopathological variability can be summarized as follows: (I) the clinical presentation generally reflected the anatomical topography of pathology, irrespective of different underlying proteinopathies; (II) hemispheric asymmetry appeared to shape clinical expression, sometimes contributing to presentations that diverged from canonical frontotemporal patterns; (III) coexisting pathologies may contribute to more complex trajectories over time, particularly in older individuals. Taken together, these patterns help explain why similar clinical syndromes may emerge when different proteinopathies affect the same neural networks, whereas less typical or asymmetric pathological involvement may lead to misleading clinical presentations (Borrego-Ecija et al., 2024; Caroppo et al., 2015). Another recurring feature is the coexistence of multiple proteinopathies, an increasingly recognized finding at autopsy that may contribute to clinical variability of dementia (Gefen et al., 2018; Karanth et al., 2020; Nelson et al., 2023). Experimental studies suggest that misfolded proteins, such as amyloid-β, tau, pTDP-43, and α-synuclein, may interact through cross-seeding mechanisms, amplifying neurodegenerative processes beyond the effect of a single dominant pathology (Nonaka et al., 2018). These observations underscore the central role of neuropathological examination in establishing the etiological diagnosis of neurodegenerative diseases. Despite advances in neuroimaging and biomarkers, *in vivo* prediction of the underlying proteinopathy remains limited in atypical and mixed cases. Brain banking protocols based on unilateral hemispheric sampling may underestimate the contribution of hemispheric asymmetry (Vonsattel et al., 2008), whereas bilateral neuropathological analysis may provide a more complete view of clinicopathological heterogeneity (Bell et al., 2008; Poloni et al., 2020).

## 5 Conclusion

This case series highlights the clinical, anatomical, and molecular heterogeneity of FTLD, showing that individual cases may deviate from classical clinicopathological expectations and primarily reflect lesion topography over the molecular substrate. By providing neuropathologically confirmed examples of clinicopathological mismatch, this series offers reference points for interpretating atypical frontotemporal presentations and supports a multimodal diagnostic work-up when available.

## Data Availability

The raw data supporting the conclusions of this article will be made available by the authors, without undue reservation.
